# A comparative evaluation of ChatGPT and Gemini for stroke caregiver support: accuracy, safety, and response characteristics

**DOI:** 10.1590/1806-9282.20251488

**Published:** 2026-06-15

**Authors:** Osman Karaca, Mustafa Savaş Torlak, Ayşe Nur Tekin

**Affiliations:** 1 KTO Karatay University, Department of Physiotherapy and Rehabilitation – Konya, Türkiye.; 2 KTO Karatay University, Vocational School of Health Sciences, Departments of Therapy and Rehabilitation – Konya, Türkiye.; 3 Baskent University Hospital Konya, Department of Physical Therapy and Rehabilitation – Konya, Türkiye.

**Keywords:** Artificial intelligence, Stroke rehabilitation, Natural language processing, Information sources, Patient safety

## Abstract

**OBJECTIVE:**

The use of large language models is increasing in healthcare. The aim of this study was to evaluate and compare the responses provided by ChatGPT (OpenAI) and Gemini (Google) to frequently asked questions about stroke and its treatment by caregivers, in terms of clarity, empathy/tone, actionability, information risk/safety, and accuracy.

**METHODS:**

This study used a qualitative content analysis to evaluate responses generated by large language models to stroke-related caregiving questions. Ten frequently asked questions were identified through a review of the literature on stroke caregiving. These questions were presented to the free versions of ChatGPT and Gemini, and responses from each model were recorded. The recorded responses were independently evaluated by 10 experts from neurology, physiatry, and physiotherapy using a structured Likert-scale instrument across five predefined categories: clarity, empathy/tone, actionability, information risk/safety, and accuracy.

**RESULTS:**

Generalized estimating equation analyses indicated that the overall trends in response scores did not differ significantly between ChatGPT and Gemini across all evaluated categories (p>0.05). In contrast, response score trends varied primarily in clarity and empathy/tone, with limited variation in actionability (p<0.05). Intraclass correlation coefficient values were below 0.50 for both ChatGPT and Gemini.

**CONCLUSION:**

Although both models generally performed well, question-level variations and low inter-rater reliability limit generalizability, and even small shortcomings in information risk may have important implications for stroke caregivers and, indirectly, for patients with stroke.

## INTRODUCTION

Natural language processing enables artificial intelligence (AI) systems to analyze text and generate or interpret human-like language^1^. In this context, ChatGPT (OpenAI) and Gemini (Google) are prominent examples of large language models (LLMs), implemented as AI-powered, multimodal systems^2^. If some ethical and technical issues are carefully addressed, they have the potential to serve as valuable tools for improving healthcare^3^.

Stroke was reported as a leading cause of death and disability, making both stroke prevention and post-stroke rehabilitation critically important^4,5^. After a stroke, harmony between the patient, their relatives, caregivers, and healthcare professionals plays a role in recovery^6^. The post-stroke period is crucial, and guidance from professionals helps patients and caregivers cope with stroke outcomes^7^. Heavy caregiving demands can lead caregivers to use escape or distancing strategies; tailored programs are needed to foster effective coping and protect their well-being^8^. Adequate stroke care education is essential for caregivers and may help reduce uncertainty and knowledge gaps by addressing informational and psychosocial needs^9^. Post-stroke caregivers have diverse care-related needs, while hospital-provided information is often limited or outdated; consequently, caregivers increasingly seek health information online^10^.

There is information in the literature that the use of LLMs on the Internet is increasing, and they can contribute as a facilitating factor in the rehabilitation of neurological diseases^11^. It is also stated that such programs can be beneficial for health professionals^12,13^, and relatives of patients^14^. In recent years, there has been a growing number of studies comparing the accuracy and effectiveness of ChatGPT and Gemini in responding to medical questions^15^.

Despite widespread use in healthcare, evidence on AI tools’ ability to address the psychosocial and practical needs of stroke caregivers is limited. This study aimed to evaluate and compare responses generated by ChatGPT and Gemini to frequently asked stroke-related caregiving questions in terms of clarity, empathy/tone, actionability, information risk/safety, and accuracy.

## METHODS

This study was conducted in December 2025. At the beginning of the study, browser histories were cleared, and new accounts were created for both ChatGPT and Gemini to minimize the influence of prior interactions.

Based on a literature review^16-20^, the following 10 questions reflecting key stroke caregiver needs, including risk reduction, recovery and rehabilitation, psychosocial support, home care, and social and administrative resources, were presented to both ChatGPT and Gemini, and the generated responses were collected for analysis; full responses are available from the corresponding author upon request.

1. How can we reduce the risk of having another stroke?
2. What is the likelihood of regaining the functions lost due to stroke?
3. How long does the recovery process take after a stroke?
4. How can I support a stroke survivor experiencing mood changes or depression?
5. How can I motivate a stroke survivor who has lost motivation for rehabilitation?
6. How can I prevent caregiver burnout?
7. What treatment and therapy options are available for stroke survivors?
8. How can I access physiotherapy services for a stroke survivor, and how can I tell if it is effective?
9. How can I properly assist a stroke survivor at home?
10. What disability pension or social support benefits are provided by the government for stroke survivors, and how can I apply for them?

Responses from ChatGPT and Gemini were independently evaluated by 10 blinded experts (three neurologists, three physiatrists, and four physiotherapists), each with ≥10 years of professional experience.

In the absence of a standardized assessment tool for AI-generated health information, evaluation domains were informed by established frameworks used to evaluate patient-facing health information and AI-generated medical content, emphasizing clarity, actionability, tone and empathy, potential information risk/safety, and accuracy^20-23^. Five domains were assessed using structured Likert scales: clarity, empathy/tone, actionability, and information risk/safety (all scored from 1 to 5, with higher scores indicating better performance), and accuracy (scored from 1 to 4, with higher scores indicating greater accuracy; the original scoring was revised for consistency)^22^. Clarity was defined as the extent to which the response was easily understandable by the target reader; empathy/tone as the degree to which the response addressed the emotional and psychosocial needs of stroke caregivers; actionability as the extent to which the response provided clear, concrete, and feasible recommendations that caregivers could implement; information risk/safety as the extent to which the response included potentially unsafe or misleading information; and accuracy as the degree to which the response was correct and sufficiently comprehensive to meet the caregiver's needs without requiring additional clarification or containing misleading or incorrect information. Experts received a written guideline explaining the evaluation domains and Likert-scale anchors prior to rating.

### Statistical analysis

All analyses were conducted using IBM SPSS Statistics version 22. Ordinal data are presented as medians and interquartile ranges (25th–75th percentiles). To account for correlated ratings from the same expert (n=10), separate ordinal generalized estimating equation (GEE) models were fitted for each evaluation domain (clarity, empathy/tone, actionability, information risk/safety, and accuracy), using a multinomial distribution with a cumulative logit link. Evaluator was specified as the subject variable, and model type and question ID were included as within-subject factors, with an exchangeable correlation structure.

For the information risk and accuracy domains, full-scale ordinal GEE models did not converge reliably; therefore, scores were dichotomized for sensitivity analyses (Information Risk/Safety: [4–5] vs. [1–3]; Accuracy: [3–4] vs. [1–2]). Inter-rater reliability was assessed using the intraclass correlation coefficient (ICC). According to established benchmarks, ICC values <0.50 indicate poor reliability^24^. A p<0.05 was considered statistically significant.

Ethical approval was obtained from the KTO Karatay University Ethics Committee (Approval No. 2025/038, 8 May 2025), and the study was conducted in accordance with the Declaration of Helsinki and national ethical standards. All participants provided written informed consent.

## RESULTS

This qualitative content analysis evaluated responses generated by ChatGPT and Gemini to frequently asked stroke-related caregiving questions. Median and interquartile range (IQR [25th–75th percentiles]) of the scores for the responses to each question are presented in Table 1. Median values are calculated across all categories for each question.

**Table 1:** Overall median (interquartile range) scores across all five evaluation categories for each question.

	ChatGPT		Gemini	
	Median	IQR	Median	IQR
Q1	4.00	3.00–4.00	4.00	3.00–4.00
Q2	4.00	3.00–4.00	3.00	3.00–4.00
Q3	4.00	3.00–4.00	3.00	3.00–4.00
Q4	4.00	3.00–5.00	4.00	3.00–5.00
Q5	4.00	3.00–4.00	4.00	3.00–5.00
Q6	4.00	3.00–5.00	3.00	3.00–5.00
Q7	3.00	3.00–4.00	4.00	3.00–4.00
Q8	4.00	3.00–4.00	4.00	3.00–4.00
Q9	4.00	3.00–4.00	4.00	3.00–5.00
Q10	3.50	3.00–4.00	4.00	3.00–4.00

Median values are calculated across all categories for each question. Q: Question; IQR: interquartile range (25th–75th percentiles).

Median and IQR of the scores for each category are presented in Table 2. Median values are calculated across all questions for each category.

**Table 2:** Median (interquartile range) scores and ordinal generalized estimating equation estimates by evaluation category.

Category	ChatGPT	Gemini	B (95%CI)	Std. error	Wald χ²	p
	Median IQR	Median IQR				
Clarity (1–5)	4.00 4.00–5.00	4.00 3.00–5.00	0.25 (-0.26, 0.76)	0.53	0.91	0.34
Empathy/ tone (1–5)	4.00 3.00–4.00	4.00 3.00–4.00	0.13 (-0.33, 0.59)	0.26	0.29	0.59
Actionability (1–5)	4.00 3.00–4.00	4.00 3.00–4.00	0.15 (-0.30, 0.61)	0.23	0.43	0.21
Information risk (1–5)	4.00 3.00–4.00	4.00 3.00–4.00	0.14 (-0.48, 0.76)	0.31	0.20	0.65^S^
Accuracy (1–4)	3.00 2.00–3.00	3.00 2.00–3.00	0.15 (-0.50, 0.79)	0.33	0.20	0.65^S^

IQR: interquartile range (25th–75th percentiles); B: parameter estimate; CI: confidence interval

S p-value derived from GEE sensitivity analysis with dichotomized scores, Gemini was used as the reference model in the ordinal GEE analyses.

In the category-level analysis, no statistically significant differences were observed between the two models for all evaluated categories (p>0.05) (Table 2).

Results of ordinal GEE analyses are shown for each question, combining data from all models. At question-level analyses, Q1, Q6, and Q9 in Clarity; Q4, Q5, and Q6 in empathy/tone; and Q9 in Actionability showed moderately higher scores compared with the reference question (p<0.05). In empathy/tone, Q9 showed borderline significance (p=0.05). Answers for the other questions did not differ significantly (p>0.05) (Table 3).

**Table 3:** Ordinal generalized estimating equation estimates results by question.

	Clarity		Empathy/tone		Actionability		Information risk		Accuracy	
	B	p	B	p	B	p	B	p^S^	B	p^S^
**Q1**	1.07	**0.04**	0.24	0.38	0.35	0.44	0.00	1.00	-0.21	0.81
**Q2**	0.17	0.77	0.50	0.13	0.47	0.33	-0.21	0.70	-0.62	0.43
**Q3**	0.26	0.66	0.29	0.41	0.17	0.67	0.48	0.40	-0.42	0.56
**Q4**	1.32	0.28	0.87	**0.006**	0.53	0.12	0.77	0.15	0.48	0.48
**Q5**	0.73	0.11	0.70	**0.009**	0.68	0.11	0.23	0.65	0.23	0.74
**Q6**	1.08	**0.046**	0.81	**0.01**	0.49	0.31	0.21	0.74	1.11	0.10
**Q7**	0.26	0.47	0.11	0.46	0.09	0.72	0.00	1.00	-0.62	0.30
**Q8**	0.73	0.86	0.53	0.13	0.39	0.31	0.23	0.56	0.77	0.40
**Q9**	1.37	**0.02**	0.51	0.050*	0.79	**0.03**	0.48	0.32	1.11	0.14
**Q10**	Reference									

Q: question, B: parameter estimate

S p-value derived from GEE sensitivity analysis with dichotomized scores, p

* borderline significance. Statistically significant values are denoted in bold.

Differences were most apparent in questions addressing psychological concerns and home-based patient assistance, which were more likely to receive higher evaluation scores. This pattern may reflect question-specific characteristics, as such questions inherently involve greater emotional and psychosocial components. However, ICC values were below 0.50 for both ChatGPT and Gemini, indicating poor inter-rater reliability.

## DISCUSSION

This study aimed to evaluate and compare the responses provided by ChatGPT and Gemini to frequently asked questions about stroke, its treatment, and caregiver-related concerns, in terms of clarity, empathy/tone, actionability, information risk/safety, and accuracy. Results indicated that the overall trends in response scores did not differ significantly between the language models across all evaluated categories. Response score trends differed significantly for some individual questions.

In a review study investigating the adequacy of LLMs in patient information, it was stated that LLMs have significant potential in patient information, but they contain deficiencies in terms of accuracy and readability, and also contain ethical concerns^21^. Another study evaluated five stroke-related questions on ChatGPT 3.5; while answers were generally adequate, sources were often inaccurate, indicating caution is needed^23^. Only one prior study (Neo et al.) has evaluated AI chatbot responses to questions from stroke patients and caregivers, reporting safety-, accuracy-, and relevance-related concerns despite broadly similar model performance^20^. Consistent with these findings, our study identified information risk/safety as a key limitation of AI-generated health advice, particularly for questions requiring symptom-specific safety considerations. Unlike Neo et al., who included both patients and caregivers, our study focused exclusively on caregivers, extending these safety-related concerns to caregiver-facing use. Qualitative examination of responses with lower information risk/safety scores revealed several recurring limitations. A prominent issue was the lack of personalization in the recommendations. For instance, in responses addressing daily care strategies for individuals with stroke, cognitive impairments commonly observed after stroke were insufficiently considered, potentially limiting the applicability of the advice. In other cases, recommendations implicitly assumed access to specific equipment or materials that may not be available to all caregivers. Experts noted that such assumptions could not only reduce feasibility but also inadvertently contribute to feelings of inadequacy or failure among caregivers when the suggested resources are unavailable. Overall, while both models showed similar scoring patterns, lower scores in the information risk domain suggest that some responses may still carry potential adverse consequences.

This study has several limitations. First, the findings represent a static snapshot of AI performance, as LLMs are updated frequently and responses may change over time. Second, ratings were based on expert judgment rather than comparison with a single verified clinical guideline. The low inter-rater agreement observed in this study is an important limitation and likely reflects the subjective nature of multidisciplinary expert evaluations, a challenge also reported in prior assessments of AI-generated health content^20^. Lastly, responses were evaluated by experts rather than stroke caregivers themselves; perceptions of empathy, clarity, and actionability may therefore differ from real end-user experiences. Future research should prioritize user-centered evaluations involving caregivers, the development of AI systems informed by curated caregiver-specific resources, and the exploration of hybrid models combining AI outputs with human moderation for sensitive caregiving guidance.

## CONCLUSION

Both models generally received favorable ratings across all evaluated categories and performed similarly across these domains. However, variations at the question level suggest that question content can influence response scores. Therefore, the findings should be interpreted with caution, and generalization of these results across different caregiving questions may not be appropriate. Notably, some lower scores observed in the information risk/safety category suggest that some responses may carry potential risks. In this context, the use of LLMs by caregivers of individuals with stroke may have the potential to lead to unintended adverse consequences if relied upon without appropriate clinical guidance.

## Data Availability

The datasets generated and/or analyzed during the current study are available from the corresponding author upon reasonable request.
